# A Genetic Association Study of *CCL5* -28 C>G (rs2280788) Polymorphism with Risk of Tuberculosis: A Meta-Analysis

**DOI:** 10.1371/journal.pone.0083422

**Published:** 2013-12-23

**Authors:** Mohammed A. A. Alqumber, Raju K. Mandal, Shafiul Haque, Aditya K. Panda, Naseem Akhter, Arif Ali

**Affiliations:** 1 Department of Laboratory Medicine, Faculty of Applied Medical Sciences, Albaha University, Albaha, Saudi Arabia; 2 Department of Urology, Sanjay Gandhi Post Graduate Institute of Medical Sciences, Lucknow, Uttar Pradesh, India; 3 Department of Biosciences, Jamia Millia Islamia (A Central University), New Delhi, India; 4 Department of Infectious Disease Biology, Institute of Life Sciences, Bhubaneswar, Odisha, India; Fundacion Huesped, Argentina

## Abstract

**Aim:**

The CC chemokine ligand 5 (*CCL5*), plays a key role in the inflammatory response by recruiting mononuclear cells during tuberculosis (TB) infection. Association studies of *CCL5* -28 C>G (rs2280788) polymorphism and TB risk have shown inconsistent and contradictory results among different ethnic populations. The aim of this meta-analysis is to investigate the association between *CCL5* -28 C>G polymorphism and TB susceptibility.

**Methodology:**

We performed quantitative synthesis for published studies based upon association between *CCL5* -28 C>G polymorphism and TB risk from PubMed (Medline), EMBASE web databases. The meta-analysis was performed and pooled odds ratios (ORs) and 95% confidence intervals (95% CIs) were calculated for all genetic models.

**Results:**

A total of six studies including 1324 TB cases and 1407 controls were involved in this meta-analysis. Variant allele (G vs. C: p = 0.257; OR = 1.809, 95% CI = 0.649 to 5.043), heterozygous (CG vs. CC: p = 0.443; OR = 1.440, 95% CI = 0.567 to 3.658) and homozygous (GG vs. CC: p = 0.160; OR = 5.140, 95% CI = 0.524 to 50.404) carriers did not show increased risk compare with those individual with the CC genotype. Similarly, no associations were found in the dominant (GG+CG vs. CC: p = 0.295; OR = 1.802, 95% CI = 0.599 to 5.412) and recessive (GG vs. CC+CG: p = 0.188; OR = 3.533, 95% CI = 0.541 to 23.085) models.

**Conclusions:**

Overall findings of this meta-analysis suggest that genetic polymorphism -28 C>G in *CCL5* is not associated with increased TB risk. However, future larger studies with group of populations will be needed to analyze the relationship between the *CCL5* -28 C>G polymorphism and risk of TB.

## Introduction

Tuberculosis (TB) is the most common chronic infectious disease caused by *Mycobacterium tuberculosis* (*M. tuberculosis*), leading cause of death with an estimate of approximately 1.5 million deaths worldwide annually [1], represents a major public health problem on a global scale. Nearly one-third of the world's population is thought to be affected with *M. tuberculosis* infection but only small fraction (5–15%) of population develops an active TB disease during their lifetime [2]. The occurrence of TB at different rates indicates that complex interaction of *M. tuberculosis* with environmental and host genetic differences may contribute to development of TB infection [3]. It is widely accepted that genetic variants, especially those belong to immune

system confer strongly influence susceptibility to active TB at the individual level [3], [4]. However, the immunopathogenesis of TB is still remains elusive and longstanding challenge for genetics research. Thus, it is anticipated that the identification of host genetic factors for susceptibility to TB would greatly aid the global control and therapeutic strategies of this infectious disease.

The immune response against *M. tuberculosis* is mostly determined by the active recruitment and activation of immune cells to the site of infection. Migration of immune cells, like activated monocytes/macrophages to the site of granuloma formation, which is a characteristic histological structure in TB infection, is mainly facilitated by adhesion molecules known as cytokines or chemokines [5]. It has been well establish that genes encoding chemokines and their receptors play an important role in the inflammatory response during TB infection [6]. The Chemotactic chemokine (C-C motif) ligand 5 (*CCL5*) gene is located on chromosome 17, a member of the beta (C-C) chemokine family and also known as ‘Regulated on Activation, Normal T cell Expressed and Secreted’ (RANTES). This chemokine plays an important role in the activation and proliferation of T-lymphocytes [7], [8], macrophages [9] and considered as a major chemokine involved in both the acute and chronic phase of inflammation and possibly participate in TB pathogenesis. These findings highlighted the importance of *CCL5* in antimycobacterial immunity and warrants for further investigations dealing with relevance of *CCL5* in mycobacterial infection.

Several functional polymorphisms in the *CCL5* gene have been described earlier, among them cytosine (C) to guanine (G) substitution of nucleotide -28 (C>G, rs2280788) found in the promoter region. The variant allele -28G was found to be associated with increased levels of mRNA and protein expression of *CCL5 in-vitro* [10]. Having known the functional significance of this genetic variant, it has been considered as potential susceptibility factors for TB. Till now, many case-control studies have been performed to investigate the association between the -28 C>G polymorphism and the risk of developing TB in various ethnic populations. Unfortunately, these studies have reported conflicting and contradictory results [11]–[16]. Inconsistency in results of these studies can be attributed to ethnicity of the population, sample size, and individual studies that have low power to evaluate the overall effect. The answer of these limitations is meta-analysis, which is a powerful tool for investigating the risk factors associated with genetic diseases, because it employs quantitative method to combine the data from individual studies where individual sample sizes are small and lower statistical power, and provides reliable conclusion [17], [18]. Hence, we have undertaken this meta-analysis to evaluate the association of *CCL5* -28 C>G polymorphism with risk of human TB.

## Materials and Methods

### Literature search strategy

We carried out a PubMed (Medline), EMBASE web database search covering all research articles published with a combination of the following key words: ‘*CCL5* OR RANTES’ gene (polymorphism OR mutation OR variant) AND tuberculosis or TB (last updated on August 2013). We evaluated potentially relevant genetic association studies by examining their titles and abstracts, and all published studies matching with the eligible criteria were retrieved.

### Inclusion and exclusion criteria

In order to minimize heterogeneity and facilitate the proper interpretation of our study, published articles included in the current meta-analysis had to meet all the following criteria: a) must evaluated the association between -28 C>G and TB risk, b) use a case-control design based on unrelated individuals, c) recruited pathologically confirmed TB patients and TB free controls, d) have available genotype frequency in case and control, e) published in English language. In addition to above, when the case-control study was included by more than one research article using the same case series, we selected the study that included the largest number of individuals. The major reasons for study exclusion were, overlapping of data, case-only studies, review articles, and genotype frequencies or number not reported. The flow diagram information related to the selection of studies is appended as supporting Figure S1 (PRISMA 2009 Flow Diagram).

### Data extraction and quality assessment

For each retrieved publication, the methodological quality assessment and data extraction were independently abstracted in duplicate by two independent investigators using a standard protocol. Data-collection form was used to ensure the accuracy of the collected data by strictly following the inclusion criteria mentioned above. The major characteristic abstracted from the retrieved studies included the name of first author, publication year, the country of origin, the number of cases and controls, source of cases and controls, study type, and genotype frequencies. Cases related with disagreement on any item of the data from the collected studies were fully discussed with investigators to reach a final consensus.

### Statistical analysis

In order to estimate the relation between *CCL5* -28 C>G polymorphism and TB risk, pooled ORs and their corresponding 95% CIs were calculated. Heterogeneity assumption was examined by the chi-square-based Q-test [19]. The significance level was maintained as p-value <0.05 for the Q-test and suggested a statistically significant heterogeneity among the studies. Pooled ORs were estimated either by the fixed effects model [20] or by the random-effects model [21]. Furthermore, I^2^ statistics was employed to quantify inter-study variability and larger values indicated an increasing degree of heterogeneity [22]. Hardy-Weinberg equilibrium (HWE) in the controls was measured via chi-square test. Funnel plot asymmetry was estimated by Egger's linear regression test which is a type of linear regression approach to measure the funnel plot asymmetry on the natural logarithm scale of the OR. The significance of the intercept was determined by the t-test (p-value <0.05 was considered as representation of statistically significant publication bias) [23]. A comparative evaluation of ‘meta-analysis’ programs was carried out by using uniform resource locator http://www.meta-analysis.com/pages/comparisons.html. The Comprehensive Meta-Analysis (CMA) V2 software program (Biostat, USA) was selected and utilized to perform all statistical analysis involved in this meta-analysis.

## Results

### Characteristics of published studies

A total of twenty six articles were finally achieved through literature search from the PubMed (Medline) and EMBASE web databases. All retrieved articles were examined carefully by reading the titles and abstracts, and the full texts for the potentially relevant publications were further checked for their suitability for this meta-analysis. Studies either showing *CCL5* polymorphism to predict survival in TB patients or considering *CCL5* variants as an indicators for response to therapy were excluded straightaway. Similarly, studies investigating the levels of *CCL5* mRNA or protein expression or relevant review articles were also excluded. We included only case-control or cohort design studies having frequency of all three genotype. Besides the database search, the references available in the retrieved articles were also checked for other potential articles (supporting Figure S1). After careful screening and following the inclusion and exclusion criteria, six eligible original published studies were finally considered for this study (Table 1). Distribution of genotypes, HWE p-value in the controls and susceptibility towards TB has been shown in Table 2.

**Table 1:** Major characteristics of the studies included in the meta-analysis.

Authors and Reference No.	Year	Country of origin	Study design	Genotyping method	Cases	Controls	Source of genotyping
Mishra et al. [11]	2012	India	PB	ARMS-PCR	215	216	Blood
Selvaraj et al. [12]	2011	India	PB	PCR-RFLP	212	213	Blood
Ben-Selma et al. [13]	2011	Tunisia	HB	PCR-RFLP	168	150	Blood
Sanchez et al. [14]	2009	Spain	PB	PCR-RFLP	76	157	Blood
Chu et al. [15]	2007	China	HB	PCR-RFLP	462	465	Blood
Mhmoud et al. [16]	2013	Sudan	HB	PCR-RFLP	191	206	Blood

Note: HB- Hospital based; PB- Population based.

**Table 2:** Distribution of *CCL5* -28 C>G polymorphism of studies included in the meta-analysis.

Authors and year	Controls				Cases				HWE
	Genotype			Minor allele	Genotype			Minor allele	
	CC	CG	GG	MAF	CC	CG	GG	MAF	p-value
Mishra et al. 2012	2	1	214	0.98	3	3	210	0.97	0.001
Selvaraj et al. 2011	208	4	0	0.009	211	1	0	0.002	0.88
Ben-Selma et al. 2011	90	50	10	0.23	29	89	105	0.67	0.40
Sanchez et al. 2009	141	16	0	0.05	56	14	6	0.17	0.50
Chu et al. 2007	370	84	11	0.11	328	79	5	0.10	0.28
Mhmoud et al. 2013	202	4	0	0.009	183	1	7	0.03	0.88

### Publication bias

Begg's funnel plot and Egger's test were performed to appraise the publication bias among the selected studies for meta-analysis. The appearance of the shape of funnel plots was seemed symmetrical in all the genetic models. The Egger's test was performed to provide the statistical evidence of funnel plot. The findings showed lack of publication bias among all comparison models (Table 3).

**Table 3:** Statistics to test publication bias and heterogeneity in the meta-analysis.

Comparison Models	Egger's regression analysis			Heterogeneity analysis			Model used for the meta-analysis
	Intercept	95% Confidence Interval	p-value	Q-value	P_heterogeneity_	I^2^ (%)	
G vs. C	−0.70	−10.38 to 8.97	0.84	87.7	<0.0001	94.3	Random
GG vs. CC	−1.37	−13.05 to 10.31	0.73	46.3	<0.0001	91.3	Random
CG vs. CC	−0.28	−5.48 to 4.90	0.88	31.3	<0.0001	84.0	Random
GG+CG vs. CC	0.23	−7.85 to 8.31	0.94	62.2	<0.0001	91.9	Random
GG vs. CC+CG	−0.85	−11.12 to 9.40	0.80	36.1	<0.0001	88.9	Random

### Test of heterogeneity

In order to test heterogeneity among the studies, Q-test and I^2^ statistics were employed. Heterogeneity was observed in all the models, i.e., allele (G vs. C), homozygous (GG vs. CC), heterozygous (CG vs. CC), dominant (GG+CG vs. CC) and recessive (GG vs. CC+CG) genotype model, which were included for this meta-analysis. Thus, random effects model was applied to synthesize the data (Table 3).

### Association of *CCL5* -28 C>G polymorphism and TB susceptibility

**Figure 1:**
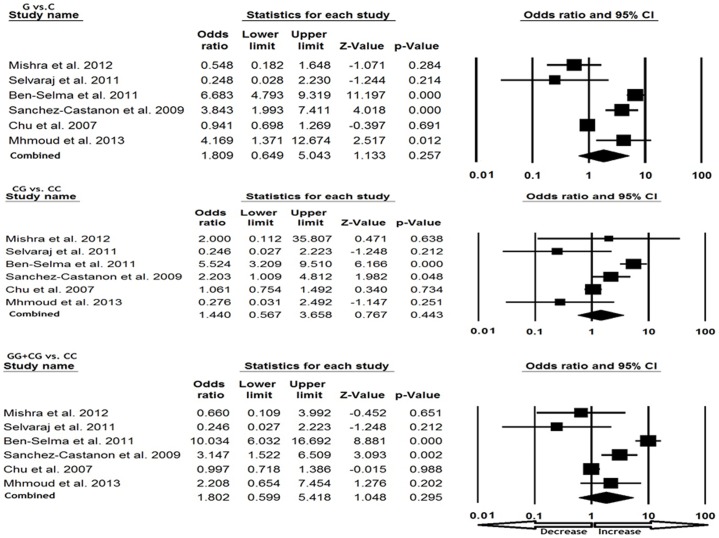
Forest plot analysis for assessing the overall TB risk associated with *CCL5* 28 C>G polymorphism. Note: The squares and horizontal lines correspond to the study-specific OR and 95% CI.

**Figure 2:**
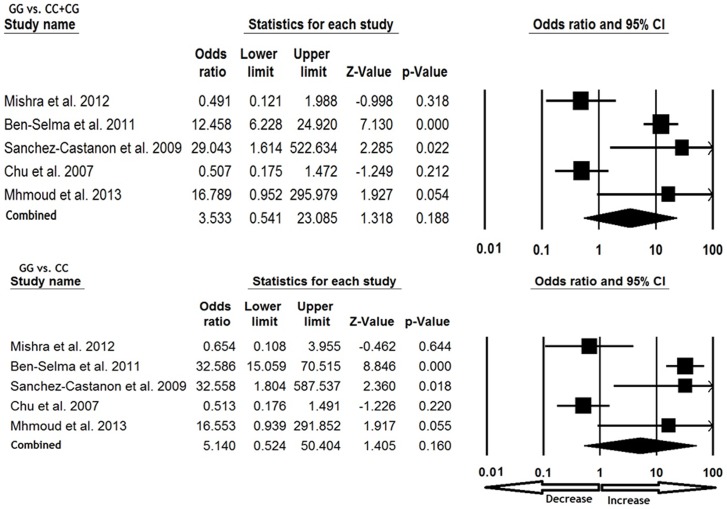
Forest plot analysis for assessing the association between TB risk and *CCL5* 28 C>G polymorphism. (Note: Analysis of GG vs. CC+CG and GG vs. CC, studies of Selvaraj et al., [12] is not included due to lack of GG genotype in both the TB cases and controls)

We pooled all six studies together and it resulted into 1324 confirmed TB cases and 1407 controls, for appraisal of overall association between the *CCL5* -28 C>G polymorphism and risk of TB. The pooled OR from overall studies indicated no significant association between *CCL5* -28 C>G polymorphism and TB risk in allelic (G vs. C: p = 0.257; OR = 1.809, 95% CI = 0.649 to 5.043), heterozygous (CG vs. CC: p = 0.443; OR = 1.440, 95% CI = 0.567 to 3.658) and homozygous (GG vs. CC: p = 0.160; OR = 5.140, 95% CI = 0.524 to 50.404) comparisons (Figure 1 and Figure 2). Likewise, dominant (GG+CG vs. CC: p = 0.295; OR = 1.802, 95% CI = 0.599 to 5.412) and recessive (GG vs. CC+CG: p = 0.188; OR = 3.533, 95% CI = 0.541 to 23.085) models also did not demonstrate any altered risk for TB (Figure 1 and Figure 2).

### Sensitivity analysis

Sensitivity analysis was performed by CMA V2 software to investigate the influence of individual study on the combined results [24]. After sequential omission of each study from the pooled analysis, the results of this meta-analysis showed no substantial change of data on all five genetic models (Figure S2). Although the genotype distributions of control groups in one study did not follow HWE. Hence, results of the sensitivity analysis suggest that the data in this meta-analysis were relatively stable and credible.

## Discussion

It is well known that TB susceptibility is determined not only by *M. tuberculosis* infection and environmental factors, host genetic factors also play an important role in the pathogenesis of this infectious disease [25]. Many polymorphic genes have been identified as TB candidate genes [26], among them *CCL5* gene plays a significant role in the antimycobacterial immune responses by recruiting mononuclear cells to the site of TB infection [5]. Chu et al. were the first to investigate the association between the incidence of TB and the *CCL5* -28 C>G polymorphism [15]. Subsequent studies demonstrated inconsistent and contradictory results, with some studies failing to find evidence of an association between the *CCL5* -28 C>G polymorphism and susceptibility of TB. Because of the above mentioned conflicting results and low power from relatively small studies, we have performed this meta-analysis with large sample size involving 1324 confirmed TB cases and 1407 controls from six studies to assess whether an association exists between the *CCL5* -28 C>G polymorphism and risk of developing TB. The pooled results demonstrated that -28 C>G polymorphism has no substantial effect on the occurrence of TB. In the same way, dominant and recessive genetic models were also not associated with risk of TB.

Chemokines play a crucial role in protective host responses during human TB infections [27]. A genetic variant -28 C>G in the promoter region of *CCL5*, has been shown to regulate the transcriptional activity of *CCL5* [10]. However, a recent study has shown that *CCL5* polymorphism is associated with lower serum level [28]. One possible explanation is that human cells have many different chemokines protein coded by genes, the majority of which are polymorphic. Thus it is possible that the analyzed variant could elevate serum level of *CCL5* may confer protection against TB. Meta-analyses of several gene disease associations have shown that initially promising associations often gravitate toward null over time [29]. Susceptibility to TB seems to be complex and variable; numerous host genes are likely to be involved in the process of active disease development [30]. Due to the multifactorial nature of TB infection and complex nature of the immune system [26], single genetic variant is usually insufficient to predict risk of this disease.

Heterogeneity between studies is very common in the genetic association studies of meta-analysis. In present meta-analysis we found inter-study heterogeneity in overall analysis. There are several factors accounting for heterogeneity, for e.g., the genetic backgrounds for cases and controls, functionally significant polymorphisms commonly differ in frequency between different ethnic groups and suggest that they are almost/always subject to natural selection [31], and inclusion of non-homogenous cases and controls. There were few limitations of our study which may influence the results minutely. First, we only included studies published in English language, abstracted and indexed by the selected electronic databases were included for data analysis; it is possible that some relevant reports published in other languages and indexed in other electronic databases may have missed. However, we did not detect publication bias. Second, the abstracted data were not stratified by other factors, for e.g., HIV status or TB severity, and these results are based on unadjusted parameters. Third, we did not test for gene and environment interactions because of the insufficient data.

## Conclusion

In conclusion, a meta-analysis is an important and reasonable approach of data-analysis which pools both statistically significant and non-significant results from individual studies and generates precise conclusions. This meta-analysis examined the relationship between *CCL5* -28 C>G polymorphism and susceptibility of TB and indicated that -28 C>G polymorphism is not associated with TB risk. Hence, screening utility of this genetic variant in asymptomatic individuals is not warranted. Nevertheless, future well designed large scale association studies including consideration of environmental factors in different populations might be necessary to improve understanding of the underlying pathophysiology and such studies might eventually lead to provide deep and precise understanding of the relationship between the *CCL5* -28 C>G polymorphism and susceptibility to TB.

## Supporting Information

Figure S1 **PRISMA 2009 Flow Diagram Flow-chart showing the overall process of study identification and selection.**
(TIF)

Figure S2 **Sensitivity analysis for** ***CCL5*** **-28 C>G polymorphism.**
(TIF)

Checklist S1 **PRISMA 2009 Checklist.**
(DOC)
