# Supplementary figures and images for: A Genetic Association Study of CCL5 -28 C>G (rs2280788) Polymorphism with Risk of Tuberculosis: A Meta-Analysis
Source: PLoS One. 2013 Dec 23;8(12):e83422. doi: 10.1371/journal.pone.0083422 (PMC3871615; doi:10.1371/journal.pone.0083422)

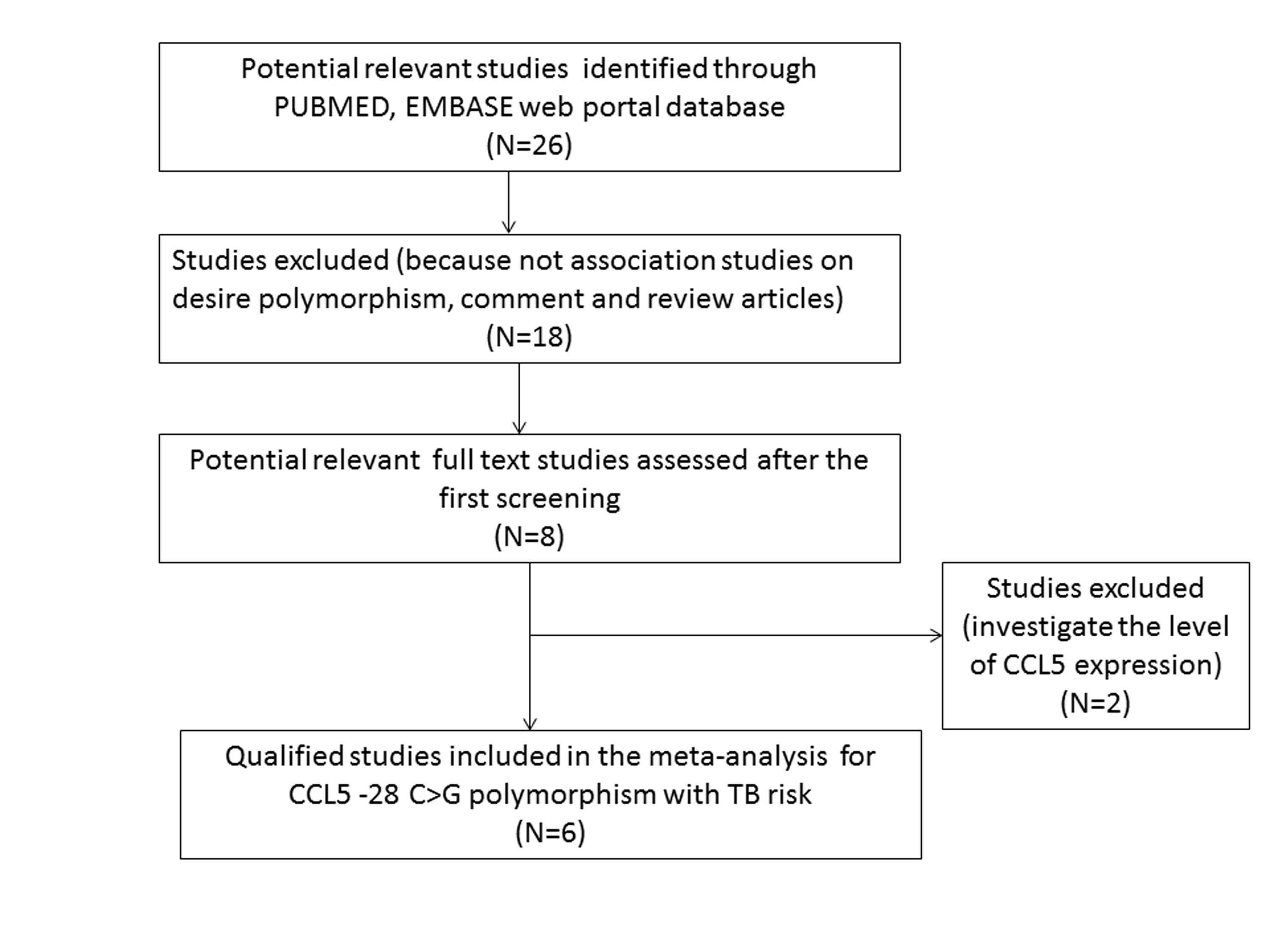

Supplement: Figure S1 — PRISMA 2009 Flow Diagram Flow-chart showing the overall process of study identification and selection. (TIF) [file pone.0083422.s001.tif]

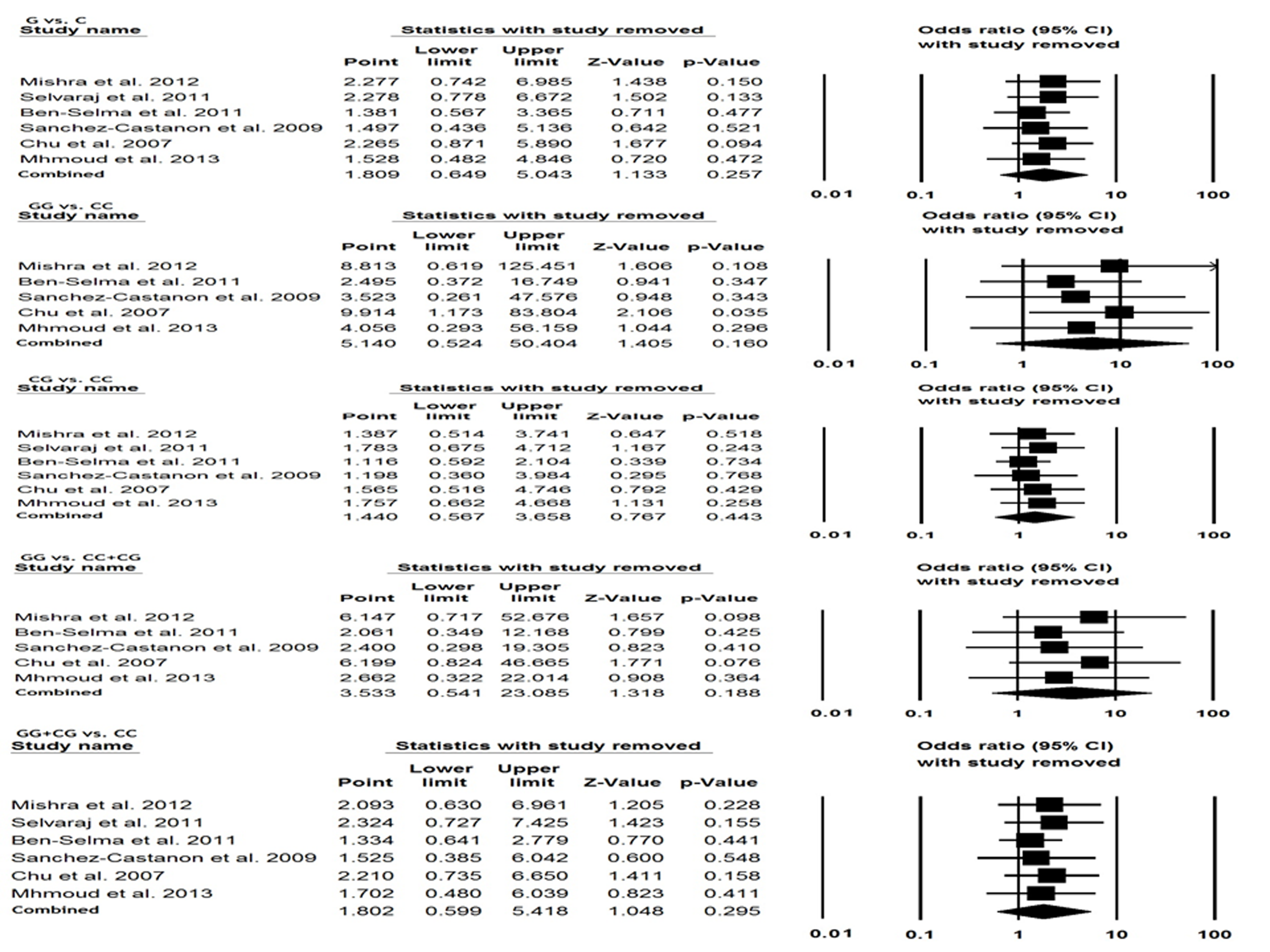

Supplement: Figure S2 — Sensitivity analysis for CCL5 -28 C>G polymorphism. (TIF) [file pone.0083422.s002.tif]
